# Linking Jasmonic Acid to Grapevine Resistance against the Biotrophic Oomycete *Plasmopara viticola*

**DOI:** 10.3389/fpls.2016.00565

**Published:** 2016-04-28

**Authors:** Ana Guerreiro, Joana Figueiredo, Marta Sousa Silva, Andreia Figueiredo

**Affiliations:** ^1^Biosystems and Integrative Sciences Institute, Science Faculty of Lisbon UniversityLisboa, Portugal; ^2^Laboratório de FTICR e Espectrometria de Massa Estrutural, Faculdade de Ciências, Universidade de LisboaLisboa, Portugal; ^3^Centro de Química e Bioquímica, Faculdade de Ciências, Universidade de LisboaLisboa, Portugal

**Keywords:** *Vitis vinifera*, biotroph, downy mildew, salicylic acid, jasmonic acid

## Abstract

Plant resistance to biotrophic pathogens is classically believed to be mediated through salicylic acid (SA) signaling leading to hypersensitive response followed by the establishment of Systemic Acquired Resistance. Jasmonic acid (JA) signaling has extensively been associated to the defense against necrotrophic pathogens and insects inducing the accumulation of secondary metabolites and PR proteins. Moreover, it is believed that plants infected with biotrophic fungi suppress JA-mediated responses. However, recent evidences have shown that certain biotrophic fungal species also trigger the activation of JA-mediated responses, suggesting a new role for JA in the defense against fungal biotrophs. *Plasmopara viticola* is a biotrophic oomycete responsible for the grapevine downy mildew, one of the most important diseases in viticulture. In this perspective, we show recent evidences of JA participation in grapevine resistance against *P. viticola*, outlining the hypothesis of JA involvement in the establishment of an incompatible interaction with this biotroph. We also show that in the first hours after *P. viticola* inoculation the levels of OPDA, JA, JA-Ile, and SA increase together with an increase of expression of genes associated to JA and SA signaling pathways. Our data suggests that, on the first hours after *P. viticola* inoculation, JA signaling pathway is activated and the outcomes of JA–SA interactions may be tailored in the defense response against this biotrophic pathogen.

## Grapevine Downy Mildew

Grapevine is one of the most valuable crops for fruit and wine production in a global scale, representing more than 7500 kHa of cultivated area in 2014 (data from the International Organization of Vine and Wine^1^). Downy mildew is one of the most economically significant grapevine diseases worldwide. It was introduced in Europe in the 1870s (Millardet, 1881) and quickly spread to all major grape-producing regions of the world (Galet, 1977; Gessler et al., 2011). The grapevine downy mildew causal agent, *Plasmopara viticola* (Berk. et Curt.) and De Toni, is a biotrophic obligatory oomycete that obtains nutrients from living cells of hosts in order to complete its life cycle. It infects all green parts of the plant, specifically leaves and clusters (Gessler et al., 2011). Under favorable conditions, motile zoospores are released from sporangia and swim toward the stomata. Subsequently, zoospores germinate and the germ tube penetrates into the substomatal cavity, primary hypha expand into the intercellular spaces of the mesophyll tissue differentiating specialized structures known as haustoria (Diez-Navajas et al., 2008). These highly specialized structures of biotrophic oomycetes and fungi play an essential role in nutrient acquisition from the plant cells and allow intense exchanges of signals that redirect the host metabolism and suppress the defense reaction (Diez-Navajas et al., 2008).

While American and Asiatic *Vitis* spp. present genetic resistance to this pathogen, domesticated grapevine *Vitis vinifera*, presently the most cultivated on a global scale, is sensitive to downy mildew. As a control measure, several fungicide applications are necessary every year and *P. viticola* resistance has already been found to the most common groups of site specific fungicides (Chen et al., 2007; Blum et al., 2010). Only in the past few decades, resistance breeding partly replaced the chemical plant protection applied against grapevine downy mildew. Partially resistant grapevine varieties resulted from breeding programs by introgression of resistant traits from wild *Vitis* spp. (e.g., *V. labrusca, V. amurensis*). However, recent reports have shown that *P. viticola* presents a high evolutionary potential as several isolates were able to break down plant resistance of interspecific hybrids (Peressotti et al., 2010; Casagrande et al., 2011). These findings have highlighted the need to fully understand grapevine resistance mechanisms against *P. viticola*.

The signaling pathways associated to grapevine and *P. viticola* interaction are still poorly understood. In plant defense against pathogens, phytohormones such as jasmonates and salicylic acid (SA) have received considerable attention (Bari and Jones, 2009). It is generally assumed that SA is involved in the activation of defense responses against biotrophic and hemi-biotrophic pathogens as well as the establishment of systemic acquired resistance, whereas inducible defense against leaf-chewing insects and necrotrophic microbes is mediated by jasmonic acid (JA)-dependent signaling (Glazebrook, 2005). These generalities are disputed in grapevine as JA signaling has been implicated in resistance against biotrophs, such as powdery and downy mildews (Hamiduzzaman et al., 2005; Belhadj et al., 2006, 2008; Trouvelot et al., 2008).

## Jasmonic Acid Signaling

**FIGURE 1:**
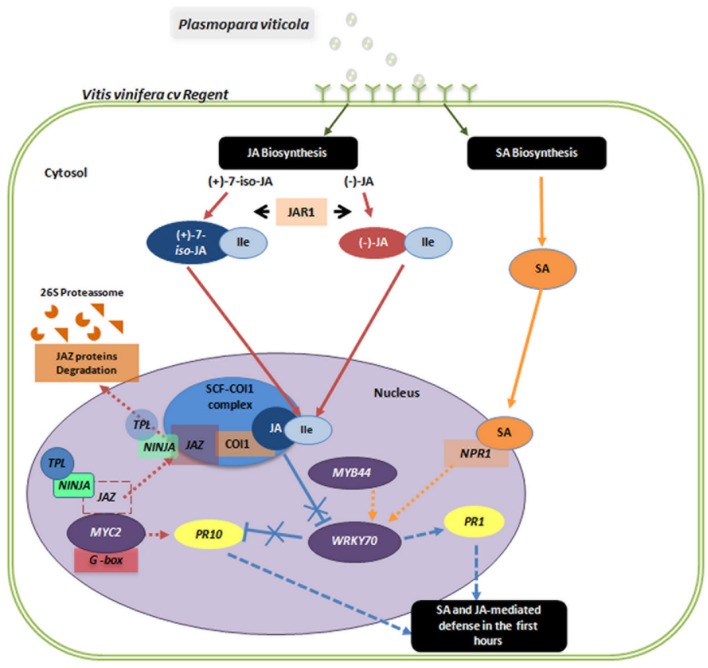
**On the first hours after *Plasmopara viticola* inoculation, the levels of SA, JA, and JA-Ile increased in *V. vinifera* cv. Regent relatively to mock inoculated control plants.** Rapid accumulation of bioactive JA-Ile promotes SCF-COI1 mediated ubiquitination and subsequent degradation of JAZ proteins and corepressors TLP and NINJA via the 26S proteasome, relieving the transcription factors such as *MYC2* and promoting the expression of JA-responsive genes such as *PR10*. High levels of SA mediate a change in the cellular redox potential, resulting in the reduction of the NPR1 oligomer to its active monomeric form. Monomeric NPR1 is then translocated into the nucleus where it functions as a transcriptional co-activator of SA-responsive genes, such as *PR-1*. Both the SA and JA signaling pathways seem to be simultaneously activated on the first hours of interaction. See text for details on the molecular processes underlying both JA- and SA-signaling. Solid lines indicate established accumulation and dashed lines suggested activities.

Jasmonic acid signaling has been extensively studied in model plants such as *Arabidopsis*. Briefly, biosynthesis of JA takes place in three different cell compartments. In the chloroplast, α-linolenic acid is released from membranes and deoxygenated by 13-lipoxygenases (13-LOXs), followed by the sequential action of allene oxide synthase (AOS) and allene oxide cyclase (AOC), resulting in the synthesis of 12-oxophytodienoic acid (OPDA). OPDA is transported to the peroxisome where the cyclopentenone ring is reduced by a *cis*-OPDA reductase 3 (OPR3) and subsequently the carboxylic acid side chain is shortened by β-oxidation to generate (+)-7-*iso*-JA, which is again released into the cytosol and epimerizes to the less active (-)-JA (Dave and Graham, 2012). In 2004, it was found that the active phytohormone is not JA itself but its isoleucine conjugate (Staswick and Tiryaki, 2004). This conjugation is catalyzed by jasmonate resistant 1 (JAR1) using (+)-7-*iso*-JA as the substrate to form bioactive jasmonate (+)-7-*iso*-jasmonoyl-L-isoleucine (JA-Ile; Staswick and Tiryaki, 2004; Fonseca et al., 2009). JA-dependent gene activation involves binding of JA-Ile to the F-box protein coronatine insensitive 1 (COI1), which acts as a JA receptor in the E3 ubiquitin-ligase SKP1-Cullin-F-box complex (SCF^COI1^). Further discovery of JASMONATE ZIM-DOMAIN (JAZ) proteins as negative regulators of JA-induced gene expression and as the true targets of SCF^COI1^ complex represented a major breakthrough in analysis of JA signaling (Chini et al., 2007; Thines et al., 2007; Yan et al., 2007). In the absence of the JA-Ile, JAZ proteins block basic helix-loop-helix leucine zipper transcription factor (MYC2) activity by recruiting the general corepressors TOPLESS (TPL) and TPL-related proteins through an interaction with the adaptor protein Novel Interactor of JAZ (NINJA; Pauwels et al., 2010). In response to JA-Ile, JAZ proteins are targeted by SCF^COI1^ for degradation, MYC2 is released activating JA-dependent gene expression and ultimately activating the regulation of various physiological processes (**Figure 1**). This model and the role of other proteins in JA perception and signaling has been widely discussed in many reviews (e.g., Wasternack, 2007; Avanci et al., 2010; Dave and Graham, 2012; Pieterse et al., 2012; Wasternack and Hause, 2013).

## Linking Jasmonic Acid Signaling to Grapevine Resistance to *Plasmopara viticola*

The first cues of JA role in grapevine resistance to downy mildew emerged from elicitor-based studies where it was shown that following both β-aminobutyric acid (BABA) and sulfated laminarin (PS3) application, the expression of LOX and JA responsive genes increased (Hamiduzzaman et al., 2005). Other studies also reported, that after *P. viticola* inoculation, the expression of *AOC* and *AOS* (Polesani et al., 2010), *LOXO* and *JAZ* (Marchive et al., 2013) and *JAZ1* and *AOC* increased (Gauthier et al., 2014). Other evidences pointing to the involvement of JA pathway came from the studies of Polesani et al. (2010) that showed an increase of JA and MeJA levels after inoculation, of Ali et al. (2012) that pointed out an increased α-linolenic acid content in resistant grapevine cultivars and Gauthier et al. (2014) that reported an transient increase in JA levels in b-1,3 glucan laminarin elicited plants.

Very recently, Figueiredo et al. (2015) characterized gene expression profile for the first steps of JA biosynthesis (*LOX2*, *AOC*, *AOS*, and *OPR3*), activation (*JAR1*) and signaling (*COI1*) in two *Vitis vinifera* cultivars with different degrees of resistance to *P. viticola*. These authors have shown that, following *P. viticola* inoculation, there was an early (6 and 12 hpi) up-regulation of JA biosynthesis-related enzymes (*LOXO, AOS, AOC*, and *OPR3*) and a later activation (18 and 24 hpi) of two of the key components of JA signaling, *JAR1* and *COI1* in the resistant cultivar. Simultaneously, an up-regulation of *LOX*, *JAZ*, and *PR14* genes and a higher content of JA (at 12 and 24 hpi) and SA (at 24, 48, and 72 hpi) was described for the incompatible *Vitis amurensis* cv. *‘Shuanghong’*–*P. viticola* interaction (Li et al., 2015).

Altogether these studies highlighted the potential role of JA in this particular plant-biotrophic pathogen interaction. To further investigate this hypothesis we have determined OPDA, JA, JA-Ile, and SA levels and conducted a qPCR expression analysis of JA-signaling associated genes [*MYC2*, *JAZ1*, and *JAZ3*, *TOPLESS*, *NINJA*, and *PR10* (pathogenesis-related protein 10)], SA-signaling markers [*NPR1* (non-expressor of PR1); *PR1* (pathogenesis-related protein 1)] and genes involved in the crosstalk between SA and JA signaling [*WRKY70* and *MYB44* (MYB domain protein 44)]. The *V. vinifera* cv. Regent, bred at the JKI-Institute for Grapevine Breeding Geilweilerhof (Akkurt et al., 2007) presenting a high degree of resistance to both downy and powdery mildew (Anonymous, 2000) was chosen as a model. Early inoculation time-points (6, 12, and 24 hpi) were considered in order to account for signaling events related to pathogen recognition in *V. vinifera*. Briefly, between 6 and 12 hpi stomatal penetration and development of stomatal vesicles with primary hyphae occur and at 24 hpi elongated hyphae invade the intercellular space of the mesophyll progressing to the branching stage in susceptible plants and stopping the development in resistant plants (Kortekamp and Zyprian, 2003; Unger et al., 2007).

**FIGURE 2:**
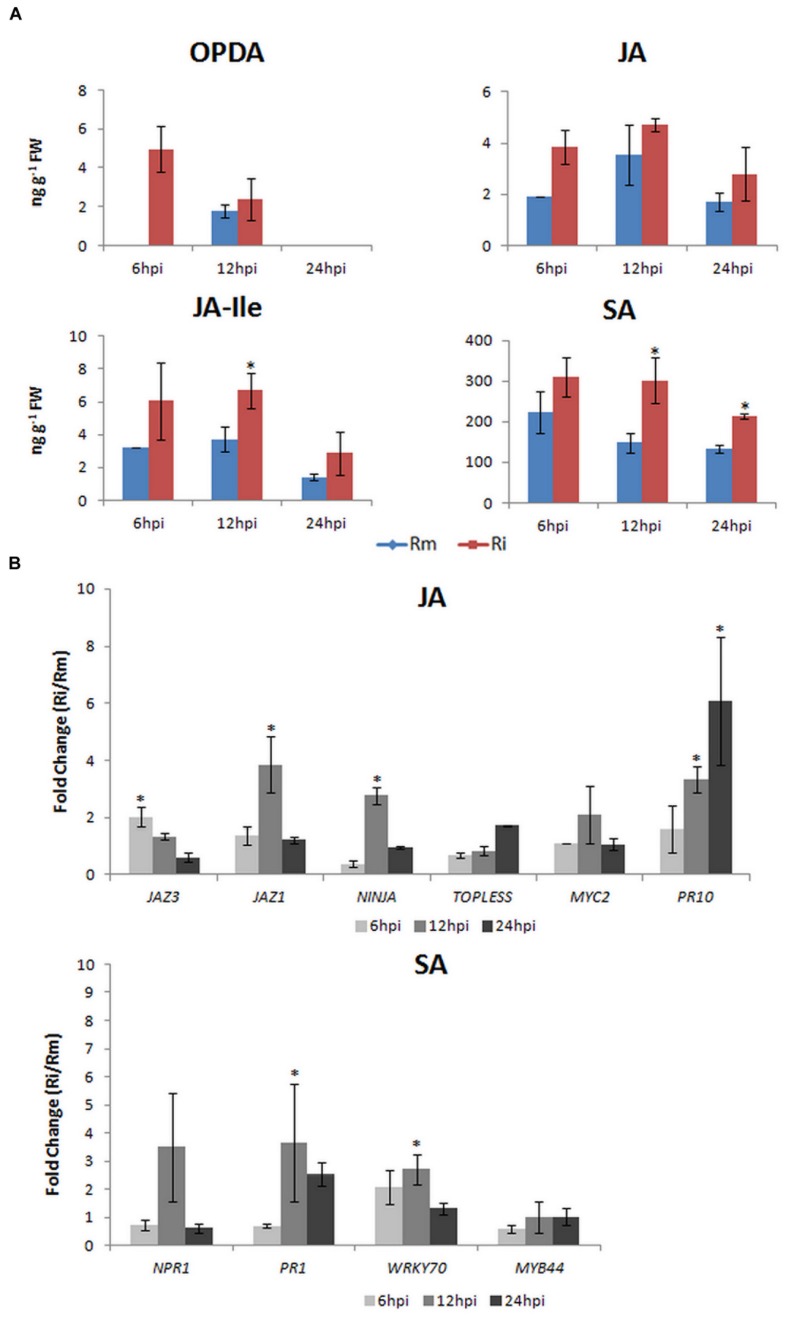
***Vitis vinifera* cv. Regent plants were inoculated with *P. viticola* (Ri) as described in Figueiredo et al. (2012).** Plant material was harvested at 6, 12, and 24 hpi. Mock inoculated samples (Rm) were done for each time-point. **(A)** Determination of the endogenous levels (ng g^-1^ FW) of OPDA, JA, JA-ILE, and SA. Briefly, 50 mg of lyophilized samples were used for phytohormone quantification in a 4000 QTRAP LC/MS/MS system (AB Sciex) at the Proteomics & Mass Spectrometry Facility at the Danforth Plant Science Center (USA). Phytohormone levels are represented as the mean and standard deviation of three biological replicates. **(B)** qPCR expression analysis of JA- and SA-signaling associated genes. Total RNA extraction, cDNA synthesis, and qPCR experiments were done according to Monteiro et al. (2013). Primer sequences, amplicon size, amplification efficiency, annealing and melting temperatures for each gene studied are given in Supplementary Table 1. To normalize expression data, ubiquitin conjugating enzyme (*UBQ*), Elongation factor 1α (*EF1α*) and glyceraldehyde-3-phosphate dehydrogenase (*GAPDH*) were used (Monteiro et al., 2013). Transcript abundance of inoculated samples relative to mock inoculated controls at each time point is represented as the mean and standard deviation of five biological replicates. Expression between 0 and 1 represents a down-regulation. Asterisks (*) represent significant difference (*p* ≤ 0.05) between inoculated and control samples at the same time point (Mann–Whitney *U* test; SPSS Inc., USA, V20).

After *P. viticola* inoculation, both *JAZ* genes analyzed also increased their expression at 6 hpi (JAZ3: 2.03 ± 0.33) and 12 hpi (JAZ1: 3.85 ± 0.98), the co-repressor TOPLESS and NINJA also increased their expression at 6 hpi (NINJA: 2.77 ± 0.29) and 24 hpi (TOPLESS: 1.72 ± 0.01). These results are coherent with the release of JAZ-bound transcription factors resulting in the activation of downstream JA responses (**Figure 2B**) and with the feed-back loop model where *de novo* synthesis of JAZ repressors is described for a negative feedback control. Moreover, in the interaction of *V. amurensis* with *P. viticola*, Li et al. (2015) have also shown an up-regulation of JAZ related genes from 24 hpi and after JA-elicitor treatment Gauthier et al. (2014) have reported an up-regulation of JAZ1 at 12 hpi.

At 12 hpi with *P. viticola MYC2* expression increased (6 hpi: 1.10 ± 0.01; 12 hpi: 2.48 ± 1.00; 24 hpi: 1.06 ± 0.20), together with the expression of *PR10* (6 hpi: 1.59 ± 0.83; 12 hpi: 3.35 ± 0.45; 24 hpi: 6.19 ± 2.24), suggesting an activation of JA signaling. This activation is corroborated by the increase of JA at 6 and 12 hpi and by the significantly increase of JA-Ile levels at 12 hpi (**Figure 2A**). After *P. viticola* inoculation it was also reported a significant increase of JA levels at 12 and 24 hpi (Li et al., 2015) in *V. amurensis* and a later increase (48 hpi) of both JA and MeJA levels in *V. riparia* (Polesani et al., 2010). *PR10* levels were also shown to increase after *P. viticola* inoculation in both Benzothiadiazole-primed and control *V. vinifera* plants (Dufour et al., 2013) and in *V. vinifera* cv. Regent plants (Figueiredo et al., 2012). Altogether, our results on this pathosystem suggest that in the resistant *V. vinifera* cv. Regent, an increase in α-linolenic acid content occurs after *P. viticola* inoculation (Ali et al., 2012) which is used for the biosynthesis of JA. The conversion of JA to its bioactive form JA-Ile is corroborated by both the increase of *JAR1* expression, described by Figueiredo et al. (2015), and the increase of JA-Ile levels at 12 hpi (**Figure 2A**). The activation of JA-dependent defense responses is suggested by the increase of *MYC2* and *PR10* expression.

## SA and JA Crosstalk in the Initial Hours of Interaction

It is generally accepted that SA activates resistance against biotrophic pathogens, while JA is critical for activation of defense against herbivorous insects and necrotrophic pathogens. Both signaling pathways are interdependent and although most reports indicate a mutually antagonistic interaction between SA- and JA-dependent signaling, synergistic interactions have been described as well (reviewed in Pieterse et al., 2012).

Signaling downstream of SA is largely controlled by the regulatory protein NPR1 that acts as a transcriptional co-activator of a large set of defense related genes, namely PR proteins (Dong, 2004) of which *PR-1* is often used as a robust marker for SA-responsive gene expression (Pieterse et al., 2012). WRKY transcription factors are important regulators of SA-dependent defense responses (reviewed in Koornneef and Pieterse, 2008) and some of them have been implicated in SA/JA cross talk, namely *WRKY70* (Li et al., 2004). *WRKY70* positively regulates SA-mediated defenses while repressing the JA response (Li et al., 2004) and in turn is transcriptionally activated by *MYB44* (Shim et al., 2013), thus both genes may be considered integrators of the cross-talk between SA and JA in plant defense responses (**Figure 1**).

After inoculation of *V. vinifera* cv. Regent with *P. viticola*, the expression of *NPR1* increased at 12 hpi (3.44 ± 1.81) decreasing afterward, when compared to mock inoculated plants (**Figure 2B**). This peak of expression at 12 hpi is accompanied by the expression of *PR1* (6 hpi: 0.70 ± 0.08; 12 hpi: 3.67 ± 2.09; 24 hpi: 2.53 ± 0.43). The levels of SA were significantly increased at both 12 and 24 hpi (**Figure 2A**). After *P. viticola* inoculation, high *PR1* levels were also described by Dufour et al. (2013) in both Benzothiadiazole-primed and control *V. vinifera* plants. Moreover, in *V. amurensis* a significant increase in SA content was also shown to occur from 24 hpi, coordinated with an increase in *PR1* expression (Li et al., 2015). Interestingly, these authors have also reported a significant increase in JA content from 12 hpi, thus both SA and JA were significantly altered at the first hours after inoculation with *P. viticola*. Although many reports describe an antagonistic interaction between the SA and JA pathways, neutral and synergistic interactions have been described as well (Mur et al., 2006). It was shown that at low concentrations SA and JA may act synergistically and at higher concentrations the effects are antagonistic, demonstrating that the outcome of the SA-JA interaction is dependent upon the relative concentration of each hormone (Mur et al., 2006).

Although WRKY70 has been implicated in SA/JA cross talk by positively regulating SA-mediated defenses while repressing the JA response (Li et al., 2004), *MYB44* shows no altered expression and *WRKY70* is slightly regulated at 6 and 12 hpi (6 hpi: 2.08 ± 0.61; 12 hpi: 2.71 ± 0.53). The expression of *WRKY70* seems to be coordinated with an increase of NPR1 and PR1 expression at 12 hpi but it does not repress the expression of *PR10*. Altogether our results suggest that at the first hours after inoculation both SA and JA pathways seem to be activated (**Figure 1**), but an antagonistic mechanism between the two pathways may be present at later inoculation time-points. The employment of synergistic/antagonistic mechanisms may represent positive and negative feedback loops allowing the tailoring of *V. vinifera* cv. Regent response to the biotrophic oomycete *P. viticola*.

## Conclusion

To reduce the environmental impact of pesticide overuse, there is an increasing interest in the use of elicitors to induce resistance against pathogens in crop plants. Disease control measures for grapevine downy mildew are based on the preventive use of phytochemical compounds. Elicitors of grapevine immunity such as BABA or PS3 are being extensively studied as alternatives for pesticide application. Here, we have highlighted the involvement of jasmonic and SA in grapevine resistance against *P. viticola*.

Future research efforts have to be made to characterize the effectiveness of JA as an elicitor of grapevine immunity against biotrophic fungi, namely on physiological adjustments, growth, yield and reduction of disease incidence. Also, very recently the effect of the foliar application of methyl jasmonate to Tempranillo grapevines to improve wine quality was studied (Portu et al., 2015). It was shown that the phenolic composition, namely 3-*O*-glucosides of petunidin and peonidin, *trans*-*p*-coumaroyl derivatives of cyanidin and peonidin and *trans*-piceid content increase significantly. Thus exogenous application of JA and jasmonates may be not only important as elicitors of grapevine immunity but also be a simple and accessible practice to enhance grape and wine quality.

## Author Contributions

AF designed the study and planned the experiment. AG and JF performed the experiments. AF, MS, AG, and JF performed data analysis. AF and MS wrote the manuscript. All authors have read and approved the manuscript.

## Conflict of Interest Statement

The authors declare that the research was conducted in the absence of any commercial or financial relationships that could be construed as a potential conflict of interest.The reviewer HK and handling Editor declared their shared affiliation, and the handling Editor states that the process nevertheless met the standards of a fair and objective review.

## Footnotes

1 www.oiv.int
