# Supplementary material for: Linking Jasmonic Acid to Grapevine Resistance against the Biotrophic Oomycete Plasmopara viticola
Source: Front Plant Sci. 2016 Apr 28;7:565. doi: 10.3389/fpls.2016.00565 (PMC4848468; doi:10.3389/fpls.2016.00565)
Supplement: Supplementary file 1 [file Table_1.DOCX]

Supplementary Table 1: Reference genes and target gene primer sequences, amplicon length, amplification efficiency, annealing and melting temperature are represented.

| ***Gene (Accession***  ***Number)**** | **Primer sequence** | **Amplicon length (bp)** | **Amplification efficiency (E)** | **Ta (ºC)** | **Tm (ºC)** |
| --- | --- | --- | --- | --- | --- |
| **Reference genes (**Monteiro et al. 2013) | | | | | |
| *EF1α*  (EC959059) | F: GAACTGGGTGCTTGATAGGC  R:ACCAAAATATCCGGAGTAAAAGA | 164 | 1.89+0.07 | 60 | 79.16 |
| *GADPH*  (EF192466) | F:TCAAGGTCAAGGACTCTAACACC  R:CCAACAACGAACATAGGAGCA | 226 | 1.99+0.10 | 60 | 80.95 |
| *UBQ*  (EC922622) | F: GAGGGTCGTCAGGATTTGGA  R:GCCCTGCACTTACCATCTTTAAG | 75 | 1.95+0.03 | 60 | 78.86 |
| **Target genes** | | | | | |
| *JAZ 1* (XM_002272327.3) | F: CAACCCAAAGCTCAACAAAG  R: TAAGTGGGAGTGGACAAGAT | 120 | 1.98+0.04 | 60 | 76.45 |
| *JAZ 3*  ([XM_002282652.2](http://www.ncbi.nlm.nih.gov/nucleotide/359491034?report=genbank&log$=nucltop&blast_rank=1&RID=63Z6A23601R)) | F: TCCCTCCTGTAAGTCCCAAT  R: TCCCCATAAAACCATCACCT | 89 | 2.05+0.115 | 60 | 77.5 |
| *MYC2*  ([XM_002280217.2](http://www.ncbi.nlm.nih.gov/nucleotide/359474556?report=genbank&log$=nucltop&blast_rank=1&RID=63ZDDG9201R)) | F: ATGCATTGCGAGCTGTTGTG  R: TCTGCCTCGGTGTTAGTTTC | 177 | 1.89+0.04 | 60 | 79.90 |
| *NINJA*  ([XM_002283943.2](http://www.ncbi.nlm.nih.gov/nucleotide/359484555?report=genbank&log$=nucltop&blast_rank=1&RID=6408EVV901R)) | F: AAATTCGGGGGATCTGGTTC  R: TGGATTGGCATGCTCTTCAC | 188 | 1.90+0.02 | 60 | 76.18 |
| *TOPLESS*  ([XM_002268229.1](http://www.ncbi.nlm.nih.gov/nucleotide/225463555?report=genbank&log$=nucltop&blast_rank=1&RID=63ZVXBMY01R)) | F: TCGGGATGGATGATTCTACA  R: GGCAAGGCCAGTTATTCTC | 97 | 1.94+0.03 | 60 | 75.89 |
| *PR10*  (HS075818) | F:GTTTTGACTGACGGCGTTGA  R:TGGTGTGGTACTTGCTGGTGTT | 99 | 1.92+0.01 | 60 | 79.99 |
| *NPR1*  ([XM_002281439.2](http://www.ncbi.nlm.nih.gov/nucleotide/359484473?report=genbank&log$=nucltop&blast_rank=1&RID=640JGKAE013)) | F: ATGGATGCCGATGACTTA  R: TCCTTGTACCTCCTCTTCTT | 85 | 1.99+0.08 | 60 | 78.42 |
| *PR1*  ([XM_002273752.2](http://www.ncbi.nlm.nih.gov/nucleotide/359475521?report=genbank&log$=nucltop&blast_rank=2&RID=640SK58401R)) | F:AAAAATGGGGTTGTGTAGGAG  R:TGTTGTGAGCATTGAGGTAGT | 112 | 1.93+0.02 | 60 | 79.20 |
| *WRKY70*  (XM_002275365.2) | F: GCCACCATACTTGCAGAGAT  R:CAGACCCAACCATATTATTAG | 89 | 2.01+0.06 | 60 | 77.97 |
| *MYB44*  (XM_002284979.2) | F: CAACGGTTTCGGGTCATAAT  R: GTTCTCGGCACTGGTCTAT | 111 | 1.93+0.02 | 60 | 79.5 |
